# Evaluating Keratoplasty for Fuchs’ Endothelial Corneal Dystrophy: A Literature Review

**DOI:** 10.7759/cureus.33639

**Published:** 2023-01-11

**Authors:** Maria Hemaya, Monica Hemaya, Amir Habeeb

**Affiliations:** 1 Hospital Medicine, Lister Hospital, East and North Hertfordshire National Health Service (NHS) Trust, Stevenage, GBR; 2 Emergency Medicine, Lister Hospital, East and North Hertfordshire National Health Service (NHS) Trust, Stevenage, GBR; 3 Surgery, Addenbrooke's Hospital, Cambridge University Hospitals National Health Service (NHS) Foundation Trust, Cambridge, GBR

**Keywords:** cultured endothelial cells, rho kinase inhibitors, descemetorhexis without endothelial keratoplasty, descemet’s membrane endothelial keratoplasty, descemet’s stripping endothelial keratoplasty, penetrating keratoplasty, corneal oedema, deturgescence, descemet's membrane, fuchs' endothelial corneal dystrophy

## Abstract

Fuchs’ endothelial corneal dystrophy (FECD) is progressive corneal endothelium dysfunction, characterised by corneal oedema, and potential blindness if left untreated. Keratoplasty is the only definitive treatment to restore vision in FECD, with different surgical techniques being described. The corneal transplant has been described as the most commonly performed and most successful allogenic transplant globally; therefore, it is crucial to dissect it further since a large proportion of the population worldwide is likely to be impacted. We feel that an updated literature review is both very relevant and necessary at present and aim to amalgamate more recent data on the topic (including meta-analyses, systematic reviews, and randomised control trials (RCTs), among others). We acknowledge that the paucity of reliable data limits progress for FECD and that there are existing ethical complexities in performing prospective trials on patients.

Traditionally, the surgery for FECD was limited to penetrating keratoplasty (PK), yet recent developments have introduced more advanced procedures and adapted the existing ones, to provide treatment specific to the disease-affected corneal layers. The questions we will address encompass: how does the severity of FECD govern the treatment options available, what are the differences between PK and types of endothelial keratoplasty (EK), what are the expected clinical outcomes of each of these operations, what are the potential concerns with the idealistic descemetorhexis surgery, and what do we envisage for times to come? Besides this, novel minimally-invasive pharmacological techniques are now being trialled, such as Rho kinase (ROCK) inhibition and cultured endothelial cells (CECs), which may drastically improve the dependence on corneal donors. We examine and critically appraise the literature to explore the understanding of FECD, and the treatment options that exist: historically, currently, and those anticipated for the future.

## Introduction and background

Fuchs’ endothelial corneal dystrophy (FECD) is a non-inflammatory, degenerative, and progressive condition that affects the endothelium of the cornea [1,2]. It is often inherited in an autosomal dominant fashion, although spontaneous mutations can occur in some patients, even in the absence of family history [1]. Usually developing slowly over decades, asymptomatic corneal "guttae" can progress to corneal thickening and oedema; causing glare and haloes, which lead to decreased visual acuity and pain [1,3,4]. FECD can be so severe that patients suffer corneal blindness [1,2]. Whilst conservative management is of symptomatic benefit and has some positive effects, it does not result in full functional rehabilitation [3,4]. Therefore, surgical treatment in the form of corneal transplantation (keratoplasty) is more definitive [3,4]. Corneal transplantation is a crucial milestone in ophthalmology. It has been described as both the most commonly performed, as well as the most successful allogenic transplant globally [5]. In 2010, it was the only method used to restore vision in FECD [6].

Corneal anatomy and function

**Figure 1:**
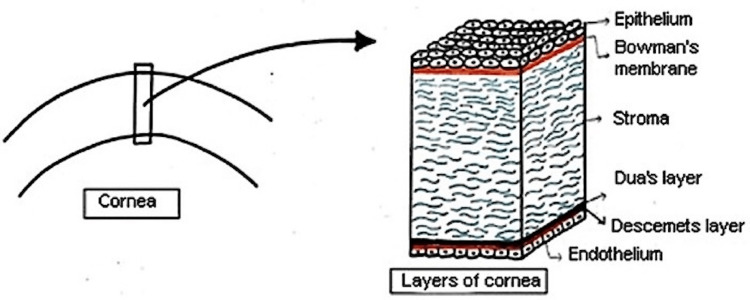
Diagrammatic representation of anatomical layers of the cornea. Sourced by open access permissions from Singh et al [5]. Drawn by the authors based on their theoretical knowledge of corneal anatomy.

The cornea is the most anterior structure of the eyeball and is avascular and transparent in nature [5]. As depicted in Figure 1, the cornea constitutes six anatomical layers: epithelium (outermost, most superficial layer), Bowman’s membrane, stroma, Dua’s layer, Descemet’s layer, and finally the endothelium (innermost layer) [5].

An anatomical distinction is important in understanding the functional properties of each layer. The epithelium comprises squamous, wing, and basal cells; it overlies the Bowman’s membrane, which exhibits regenerative qualities [5]. The third corneal layer is the stroma, which is composed of 78% water [1]. The stroma consists of keratocytes and collagen lamellae that are more densely allocated anteriorly rather than posteriorly [4,5]. Though Dua’s layer is a distinct entity, it continues to be firmly adherent to the overlying stroma, and it is nearly 10-15 μm thick [5]. The fifth layer is Descemet’s membrane (DM), which acts as a basement membrane for the final layer (the endothelium) [5,6].

The endothelium contains a monolayer of hexagonal cells, is in contact with the aqueous humour of the eye, and has the important purpose of ensuring corneal transparency [5,6]. This is achieved by acting simultaneously as a passive barrier and an active pump in tandem, allowing the endothelial cells to preserve the cornea in an environment of deturgescence (relative dehydration) [2,4,6-8]. During the active transport of solutes from the aqueous humour, water will passively enter the corneal stroma; the endothelium, therefore, is a barrier to excess fluid influx but also has an active role in pumping fluid out of the cornea via sodium-activated ATPase [2,4,6-8]. An adequate number of endothelial cells are needed for appropriate deturgescence; otherwise, the pump function breaks down and the cornea becomes oedematous [8].

Pathophysiology

In the initial phases, FECD features endothelial cell loss and altered hexagonal mosaic morphology, together with accumulation of extracellular matrix deposits in the DM called "guttae" or "excrescences" or "warts" [1-2,4,6,9]. These excrescences are small protuberances, frequently shaped like mushroom caps on the endothelial surface [1]. Guttae formation and endothelial cell loss begin centrally and then spread to the peripheral cornea [1-2,8]. Both variations in cell size and cell shape are seen; they are termed polymegathism and pleomorphism, respectively [2,4]. Some of these changes are pictured in Wilson et al.’s light micrographs and specular microscopy [4].

Over time, since the natural watertight seal of the endothelium has been undermined, the anterior chamber fluid aggregates in the stroma so that it becomes too saturated and waterlogged [1,7]. This increases the stromal thickness, causes light to scatter, and means blurred vision is a potential consequence [1]. Patients may suffer morning misting since evaporation at the surface cannot happen whilst the eyes are closed, so the cornea swells overnight [1,7]. This cloudy vision often clears towards the end of the day, owing to more tear evaporation causing a higher tear osmolality, which draws water out of the tissue, and therefore reduces corneal oedema [1,4,7]. Similarly, patients may notice worse vision in more humid or rainy weather but improved when it is dry [1]. In addition, they may experience reduced contrast sensitivity, glare and coloured haloes around bright objects, or problems with driving at night [1,4]. 

As FECD advances, oedema also collects in the epithelium, resulting in microcysts and "bullae" (large blisters) that render the epithelial surface uneven and corrugated [1,4]. These bullae can tear or rupture, hence causing painful corneal erosions and open wounds that are a portal for infection [1,4]. Thus, dense corneal oedema and bullous keratopathy may be present in severe FECD [1]. Chronic oedema can trigger the formation of subepithelial fibrosis, thereby contributing to opacity in the cornea, which can eventually lead to permanent scar tissue and the development of pannus (corneal vascularisation) in an otherwise avascular structure [1]. 

Some sources have linked FECD to oxidative DNA damage and apoptosis, especially in mitochondria, suggesting a potential area of research for future treatments [2,9]. The corneal endothelium is considered particularly susceptible to oxidative stress because of its continual exposure to light reaching the retina, its high oxygen demand from its copious metabolism of ions via ATPases, and also postmitotic arrest [2]. Jurkunas et al. have further illustrated the pathogenesis of FECD due to these aspects in a diagram [2]. Although these mechanisms are still not fully understood, it is generally accepted that FECD involves an interplay between genetic and environmental factors and has a greater incidence in women, typically in their fourth to fifth decade of life [1-2,9].

Diagnosis

Whilst the diagnosis is mainly clinical, slit lamp examination in the early stages can reveal endothelial aberrations (e.g., guttae) and the mild stromal oedema and the guttae can be seen more easily using the red reflex, as shown respectively in Feldman et al.’s first and second clinical photos [1]. As FECD is slowly progressive, patients initially might not even recognise any visual decline; but ultimately the compound effect of stromal opacification and irregular astigmatism will exacerbate the deterioration in acuity [1,4]. Severe corneal opacification may hinder the ability to view the anterior segment and endothelium, so diagnosis is more difficult; ergo, the FECD diagnosis can be reached according to the history or by examination of the contralateral eye (since the corneal changes are bilateral but often asymmetrical) [1,4].

Other investigations may be useful in FECD. Fluorescein dye can be of dual benefit: it can pool in the indentations on the epithelial surface (accentuating the defects), but it can also emphasise the microcysts by leaving behind patches of negative staining [1,4]. Pachymetry (assessment of corneal thickness) may be utilised in monitoring disease progression and severity (as the cornea becomes thicker as FECD worsens) [1,4,10]. Specular microscopy can make the endothelial changes more evident, for instance: the guttae (appear as darkened, scattered areas), cell alterations, and low endothelial cell counts per unit area [1,4]. Furthermore, fluorophotometry measures the fluorescein concentration in the cornea, and considering fluorescein is expected to have proportional movement across membranes to water, these values can then help calculate endothelial permeability (barrier function) [4].

Management

Some patients will be treated using a watchful management approach [10]. Medical treatment of FECD includes topical hypertonic saline (e.g., sodium chloride), as this can help extract the excess water from the cornea, hence bettering vision [1,4,10]. Moreover, conservative methods that evaporate fluid off the cornea may also be beneficial, such as blowing air near the eyes from a hair dryer held at a distance [1,4]. Painful ruptured bullae can be treated using bandaged contact lenses, but this requires frequent follow-up due to the high risk of infection [1]. Regrettably, most patients are left dissatisfied with these therapy options, and if medical management fails then surgery is imperative [1,4].

**Figure 2:**
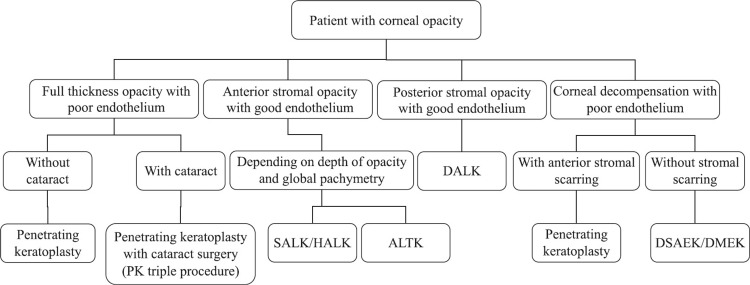
Flowchart depicting a stepwise approach while planning surgical management in a case of corneal opacity. Sourced by open access permissions from Singh et al [5]. PK: penetrating keratoplasty, SALK: superficial anterior lamellar keratoplasty, HALK: hemi-automated lamellar keratoplasty; ALTK: automated lamellar therapeutic keratoplasty; DALK: deep anterior lamellar keratoplasty, DSAEK: Descemet’s stripping automated endothelial keratoplasty, DMEK: Descemet's membrane endothelial keratoplasty. PK, DSAEK, and DMEK have already been described. Note that SALK, HALK, ALTK, and DALK are beyond the scope of this literature review.

Traditionally, the surgical management of severe FECD was penetrating keratoplasty (PK), which involved replacing the full-thickness of the cornea, and multiple sutures would hold the cornea in the correct position, as seen in Feldman et al.’s third clinical photo [1]. However, in the last two decades, techniques that only necessitate transplantation of the endothelium (posterior cornea) have been favoured, such as endothelial keratoplasty (EK) (also known as posterior lamellar surgery), which is now the standard management of early to moderate FECD [1]. Types of EK include: Descemet’s stripping endothelial keratoplasty (DSEK) and Descemet's membrane endothelial keratoplasty (DMEK), which transplants an even thinner piece of tissue, followed by the most recent technique of Descemetorhexis without endothelial keratoplasty (DWEK), also called Descemet's stripping only (DSO) [1]. The most common type of EK in the USA is Descemet’s stripping automated endothelial keratoplasty (DSAEK) [8]. Figure 2 is a flowchart showing a stepwise approach to planning surgical management in a patient with opacity of the cornea [5].

## Review

Penetrating keratoplasty

Allan’s first illustration portrays the anatomy of the eye by the sagittal cross-section [11]. In PK, the full thickness of the host cornea is dissected and replaced by full-thickness donor tissue in the shape of a disc, and sewn in position [7,11]. This is further depicted by the sagittal cross-section in Allan’s third illustration [11]. This circular wound will heal over time, and the sutures can then be removed in months or even a couple of years after the surgery [1,7,11]. In contrast to EK, surgical manipulation of the donor tissue in PK is minimal, but sutures are needed to anchor the donor tissue [12]. The outcomes for conventional PK vary, with the majority of grafts remaining clear for five years or more after surgery; yet if a graft fails (becomes cloudy) and repeat keratoplasty is performed, the probability of a favourable outcome recedes with successive grafts [7]. Factors that may contribute to graft failure incorporate: graft rejection (prophylactic topical steroid drops are usually required), infection, and glaucoma [1,7]. Furthermore, even if the graft remains clear, vision does not always improve after PK, especially because of astigmatism; hence, correction of the latter via surgery or suture manipulation may be necessary [7,13]. 

Vision can take time to stabilise, and glasses or contact lenses may be used to improve visual outcomes post-operatively [1,13]. Overall, although PK is often successful and can restore vision despite advanced FECD, patients might undergo a relatively delayed recovery [1,13]. In 2016, one review noted that in comparison to the other keratoplasty techniques, PK had the greatest rejection rate, intra- and post-operative complications, and post-operative astigmatism [14]. In 2019, it was also described that, due to the large incisions in PK, the risk of wound dehiscence (either spontaneous or traumatic) could even lead to loss of the eye, and devastating complications could occur secondary to the sutures and impaired healing of the eye surface [15]. They proclaimed that EK has consequently been very valuable over time because of its preferable surgical outcomes [15].

Endothelial keratoplasty

In contrast to PK, the new endothelial layer in EK is transplanted on a thin layer of donor cornea and attaches with no sutures, whilst the eye wall remains intact [7]. This technique has several benefits, particularly allowing a normal corneal shape, strength, and refractive power [7]. The favourable aspects of intentionally replacing the diseased corneal layers include: preserving corneal architectonics, swift restoration of vision, "closed eye" operations via a "tunnel" method, and more independence from costly equipment [3]. DSAEK involves descemetorhexis of the host cornea and removal of the DM and affected endothelium [8]. The donor tissue used as a replacement is about 100-200 µm thick and comprises healthy endothelium, DM, and also a thin layer of posterior corneal stroma that strengthens the eye wall [1,8,11]. DSAEK is still preferred by some surgeons, as the inclusion of the supplementary stromal layer provides more support for the graft and can make the procedure easier to perform [11]. Following corneal transplantation, an air bubble is inserted in the anterior chamber to temporarily assist with graft attachment, and it dissolves within a few days [1,8]. An air bubble one day after DSEK can be seen in Feldman et al.’s fourth clinical photo, where it is filling 30 to 40% of the anterior chamber [1]. 

Unlike traditional PK, DSEK can be executed via a significantly smaller incision with minimal (if any) sutures and hence less suture-induced astigmatism [1]. As a result, DSEK leads to higher quality vision, weaker prescription glasses (when required), and quicker visual recovery; whilst the smaller incision means fewer wound leaks, a reduced likelihood of infection, better eye stability, and a greater ability to withstand damage from eye trauma [1]. Similarly to PK, other ocular surgeries, e.g., cataract removal, may be completed at the same time as DSEK and DMEK [1,11]. For mild-moderate FECD, DSEK is considered superior to PK; however, if chronic swelling has led to corneal scarring, then sole endothelial transplantation is likely to be visually unsatisfactory, necessitating the use of PK [1]. One must also be mindful of the increased risk of rejection of the corneal transplant in DSAEK, secondary to the additional foreign stromal tissue, which is why DMEK is often recommended instead [11]. Yet DMEK demands more surgical skill than DSAEK, as in the absence of the adherent stroma, the elastic tissue leads to the folding of the graft into a scroll, and subsequent unfolding strategies are challenging [8]. 

DMEK boasts an even thinner corneal graft (resulting in better quality vision), where the endothelium is transplanted with its native supporting membrane alone (the DM), without stroma [1,7]. The technique uses small, self-sealing incisions at the limbus and is depicted by coronal and sagittal cross-sections in Allan’s second illustration [11]. It was developed in 2006 by Dr. Gerrit Melles and was seen as a minimally invasive operation with quicker recovery times in comparison to DSEK [1,13]. Like DSEK, DMEK also implants an air bubble to enable adherence of the transplanted tissue, with the patient remaining supine (face up) for a few days post-operatively [1]. However, since thinner tissue is used in DMEK, concerns include that graft attachment may present with more challenges, a prolonged duration may be required in the supine position, or even more "rebubbling" procedures may be needed than for DSEK [1]. DMEK may be more arduous than DSAEK; firstly because of the issues in preparing the donor tissue without wastage, and secondly during insertion, manipulation, and adhesion of the delicate donor endothelium [10,12]. One published review highlights that the adoption of DMEK in the past has been slower than DSEK due to these increased technical difficulties as well as the scarcity of evidence demonstrating its superior clinical outcomes over DSEK [12]. This review further noted that DMEK has a high rate of primary iatrogenic graft failure and proposed that for DMEK to be more successful, endothelial cell loss and graft survival would need to be analogous or preferable to those following DSEK [12]. Patel shows a schematic representation of different types of keratoplasty for endothelial disease (including PK, DSEK, and DMEK) [12].

Descemetorhexis

Finally, we describe DWEK or DSO. This is especially indicated for FECD patients with central corneal guttae or the "central phenotype," but a relatively unaffected peripheral endothelium [1,8,11]. The DWEK surgery is identical to the initial stage of DMEK; however, it exhibits descemetorhexis alone, as the afflicted 4-5 mm of central DM is excised but no donor cornea is transplanted [1,11]. It relies on healthy peripheral endothelial cells migrating centrally, thereby restoring the pump function and therefore vision [1,11,16]. Since the endothelium must repopulate sufficiently to restore corneal deturgescence, the time to visual recovery is longer after DWEK [8]. Hence, DMEK is more suitable for patients that cannot tolerate prolonged periods of decreased vision, as it is characterised by quicker corneal clearance [8]. Bruinsma et al.’s diagrams portray the regenerative potential of corneal endothelial cells in response to oxidative stress, as well as the central migration of these cells via spontaneous clearance and descemetorhexis alone [16]. 

Rho kinase (ROCK) inhibitor ocular drops (such as ripasudil) may be given following DWEK as an adjunct, as they can stimulate the movement of the endothelial cells (these drops are licensed for glaucoma in Japan, but not yet routinely available in the UK, nor currently approved in the US for this context) [1,11]. ROCK is the downstream effector of RhoA, a GTPase protein that is a member of the Ras family [13]. This RhoA/ROCK pathway allows regulation of cell migration, proliferation, and apoptosis and has thus been a great point of interest in treating endothelial diseases, particularly in early-stage dysfunction [13,17]. There are numerous advantages to DWEK, chiefly in that there is no graft rejection (as no corneal tissue is transplanted) and likewise no requirement for long-term surveillance when taking steroid drops [1]. Besides these, the wound is very small, and patients do not have to be positioned supine after the procedure [1]. DWEK also has a notably high rate of success [8]. Yet there are some concerns, for instance, a temporary worsening of vision shortly after the operation in light of central corneal oedema [1]. As well as the slower recovery of DWEK, if unsuccessful, patients may ultimately require DMEK or DSEK surgeries [1,8,11]. Therefore, DWEK can be contemplated for younger individuals with mild central FECD, nevertheless, it is not yet championed as a routine treatment option [13]. It is important to appropriately counsel patients about this and recognise that, as DWEK is a newer technique, data from long-term follow-up is more limited [8].

Evidence-based discussion

In 2018, a single-centre study of prospectively collected data from the national database of corneal transplant follow-up measured 10-year graft survival and visual function following three surgical techniques for FECD (including conventional PK and DSAEK) [18]. There were 171 conventional PK cases included, and 459 DSAEK cases, yet they found that there was no significant difference in graft survival between the two [18]. Best-corrected visual acuity (BCVA) was calculated using the logarithm of the minimum angle of resolution (logMAR) scale. This study noted better pre-operative BCVA in the DSAEK category (0.68 ± 0.41 logMAR) than in the PK category (0.89 ± 0.53), as well as significantly better post-operative BCVA after 24 months in the DSAEK group (0.25 ± 0.26 logMAR) than in the PK group (0.35 ± 0.29), P < 0.001 [18]. This suggested that the DSAEK candidates had been selected at an earlier FECD stage than the PK candidates [18]. If PK candidates had a more severe stage of FECD from the outset, then this restricts the conclusions drawn, as the pre-treatment baseline was inconsistent between the two techniques. In this study, however, the authors concluded there was no difference in the BCVA improvement between DSAEK and PK [18]. Reporting the relative change rather than absolute values, therefore, allows a fairer comparison of these surgeries.

Nonetheless, they found significantly lower induced astigmatism after 24 months in the DSAEK group (1.7 ± 1.1 dioptres) versus the PK group (4.6 ± 2.7 dioptres), confirmed by vector analysis [18]. The other factors favouring DSAEK over PK included: greater wound stability and faster visual rehabilitation, suggesting earlier intervention with DSAEK is warranted to help preserve vision [18]. The major strengths of this study are that it incorporated a higher caseload than the PK versus EK trials discussed below, the keratoplasties were performed by four experienced surgeons, and the researchers observed clinical outcomes for a long period after treatment (a decade). It was described by the authors as a retrospective study with structured prospective follow-up visits [18]. However, it is limited since it was a single-centre, non-randomised study, and any exclusion criteria were not clearly defined. 

A Cochrane systematic review in 2014 compared general EK surgery for FECD with PK, searching databases such as CENTRAL, MEDLINE, EMBASE, the meta-register of controlled trials, and www.clinicaltrials.gov [19]. They incorporated the data from three RCTs, initially enlisting 139 eyes from 136 participants, but then analysing 123 eyes [19]. Their selection criteria were clearly stated. Two of the RCTs randomly allocated eyes to one of the EK or PK groups, alongside the third RCT, which randomised eyes into either the femtosecond laser-assisted EK (FLEK) category or the PK category [19]. The former RCTs did not demonstrate any significant differences between the BCVA of EK and PK surgeries at two years or one year, yet the latter trial showed significantly better BCVA after PK rather than FLEK (mean difference 0.20 logMAR, 95% confidence interval (CI) 0.10 to 0.30, P = 0.0001) [19]. Only one of the former RCTs described irregular astigmatism, observing that it was less following EK than PK (mean difference −1.20 µm, 95% CI −1.53 to −0.87, P < 0.001), similar to the 2018 study above on DSAEK and PK [18,19]. 

The latter RCT on FLEK versus PK ascertained that there were lower endothelial cell counts (indicating greater cell loss due to the procedure), higher cases of primary graft failure (8% and 0%, respectively), and more graft rejection (3% and 2%, respectively) in the FLEK group than the PK group [19]. Likewise, this RCT reported that the FLEK group demonstrated graft dislocation in 27.8% of participants, intraocular pressure-related problems in 13.9%, and epithelial ingrowth and post-operative pupillary block in 2.8% [19]. Conversely, the PK group in this RCT showed suture-related complications in 10%, required revision of sutures to correct astigmatism in 10%, and experienced wound dehiscence in 5% of participants [19]. Overall, this Cochrane systematic review deemed that the overall quality of methods in the three RCTs was unsatisfactory, observing particularly the lack of allocation concealment, lack of participant and assessor masking (performance bias), as well as the small sample size of all RCTs [19]. Two studies were deemed at high risk of attrition bias, one due to the high proportion of losses to follow-up, and the other had a treatment group change without a clear reason why [19]. Another challenge to highlight is that multiple types of EK (e.g., DSEK, DSAEK, DMEK, and FLEK) were grouped in these RCTs, which may have a substantial impact on the overall results as they each have significant variations in technique and complication rates [19]. Evidently, there is a paucity of large, unbiased, high-quality randomised-control data on visual outcomes and long-term graft survival following differing keratoplasty techniques for FECD, and future trials may wish to also investigate the quality of life, vision, outcomes after long-term follow-up, risks of endothelial rejection, as well as cost-effectiveness [19]. 

Interestingly, in 2021, a retrospective comparative cohort study of FECD patients did in fact examine the quality of life and visual outcomes after a prolonged follow-up period [20]. Their cohort included 13 patients who had undergone bilateral ultrathin DSAEK, with 11 patients who had bilateral PK; all of whom were already pseudophakic or had sustained a dual keratoplasty and cataract surgery [20]. Following the second eye procedure, the mean follow-up was 19.6 ± 8.6 months in the DSAEK group and a more impressive 32.5 ± 10.2 months in the PK group [20]. The corrected-distance visual acuity was significantly better in the DSAEK group versus the PK one (0.18 ± 0.07 compared to 0.35 ± 0.16 logMAR, p < 0.0001) [20]. Although the mean posterior cornea total higher-order aberrations (HOAs), which are more complex distortions did not differ between the two cohorts, the mean anterior total HOAs (of the central 5 mm zone) were significantly less after DSAEK than PK (0.438 ± 0.078 µm and 1.282 ± 0.330 µm respectively, p < 0.0001) [20]. 

Alongside these preceding findings, the contrast sensitivity was also greater in DSAEK eyes than in PK eyes, allowing the authors to confirm that DSAEK demonstrated better visual function overall [20]. Consequently, the vision-related quality of life, which was binocularly evaluated using the scores from the National Eye Institute Refractive Error Quality of Life Instrument-42 (NEI RQL-42) test, indicated higher satisfaction in DSAEK patients (in nine out of 13 scales, to be specific) [20]. Fortunately, statistical analysis (incorporating descriptive) of the quantitative data was applied to all the results, for instance, covariance analysis was utilised to adjust for any pre-existing discrepancies in non-equivalent categories (where the random assignment was unable to make the groups equal) [20]. The Fischer exact or chi-squared tests were performed to enable comparison between categorical variables [20]. 

Since there has been a general shift from PK with a corresponding rise in EK procedures in recent decades, reports in the literature have critiqued the different EK types. For example, a meta-analysis published in 2018 examined the safety and efficacy of DMEK and DSAEK in adults with FECD by searching MEDLINE and CENTRAL electronic databases from the outset to August 2017, counting all comparative studies of the two aforementioned operations [21]. In an attempt to curtail bias, any research investigating rescue procedures was excluded [21]. Both primary outcomes (post-operative BCVA after three, six, and 12 months) and secondary outcomes (primary graft failure, rejection, "rebubbling," endothelial cell density, subjective vision, and patient satisfaction) were examined [21]. This meta-analysis consisted of 10 retrospective studies of moderate-quality methodology, containing 947 eyes in total (646 DMEK and 301 DSAEK), where five trials of 164 eyes were comparative bilaterally (82 patients) [21]. They concluded that BCVA was superior following DMEK as opposed to DSAEK at all the measured time scales (0.16 logMAR at 12 months and 0.30 logMAR, p < 0.001, respectively) [21]. Moreover, DMEK displayed a 60% lower rejection rate (risk ratio = 0.4, 95% CI (0.24, 0.67), p = 0.0005) and greater patient satisfaction (odds ratio = 10.29, 95% CI (3.55, 29.80), p < 0.0001), albeit more "rebubbling" (risk ratio = 2.48, 95% CI (1.32, 4.64), p = 0.005) [21]. DMEK also exhibited more primary graft failure, and lower endothelial cell density loss; however, these differences were not statistically significant [21]. Once again, caution should be exercised with these results, given the small number of trials and short follow-up times (despite the reasonably high caseload). 

Congruous figures were reached by a retrospective contralateral study of 10 patients, comparing DMEK in one eye with DSAEK in the other eye [22]. BCVA after DMEK was significantly better than after DSAEK (0.16 ± 0.10 versus 0.45 ± 0.58 logMAR, P = 0.043, respectively) [22]. In addition, the contrast threshold (a measure of contrast sensitivity) was significantly higher following DMEK than DSAEK (0.49 ± 0.23 versus 0.25 ± 0.18, P = 0.043, respectively) [22]. There were minimal differences between the two in terms of post-operative astigmatism, mean spherical equivalent, HOAs, visual outcome, post-operative pain and burden, and the estimated time for recovery and rehabilitation [22]. Using a subjective questionnaire, the mean patient satisfaction was evaluated highly and equally after both operations; however, 90% of the patients preferred DMEK if given the choice (perhaps due to the better visual acuity and contrast sensitivity) [22]. To overcome the inability to determine a normal distribution for all the outcome measures, the researchers applied a paired non-parametric Wilcoxon test to statistically analyse continuous variables [22]. They raised concerns that there was potential for recall bias in the patient satisfaction survey due to the various follow-up times following each surgery and proposed that more detailed prospective studies could be introduced instead [22]. 

Likewise, a 2018 Cochrane systematic review also established that DMEK can lead to better BCVA at 12 months compared to DSAEK for endothelial failure (mean difference −0.14, 95% CI −0.18 to −0.10 logMAR in 140 eyes) [23]. This systematic review examined four non-randomised trials, comprising 72 patients (144 eyes), who had undergone DSAEK in one eye, prior to DMEK in the other eye [23]. It was seen as low-certainty evidence with a high risk of bias owing to potential confounding variables (as DMEK was preceded by DSAEK in all patients) [23]. Corresponding to the above 2018 meta-analysis, this Cochrane systematic review reported more graft dislocations requiring "rebubbling" with DMEK than DSAEK (risk ratio = 5.40, 95% CI 1.51-19.3, 144 eyes), but considered this very low-certainty evidence [23]. In 2020, another retrospective contralateral analysis of nine FECD patients stated that, due to the supplementary stromal tissue, DSAEK resulted in higher total stromal backscattering (haze) than DMEK [24]. Similar to the above conclusions, BCVA was better following DMEK than DSAEK, which the authors partly attributed to the lower total stromal backscattering and lower posterior cornea HOAs in DMEK [24]. 

Finally, we appraise the outcomes and morbidity of the more recent DWEK technique against DMEK. In 2018, a retrospective comparative cohort of 27 eyes (12 DWEK and 15 DMEK) with mild to moderate FECD was published [25]. These patients were treated from 2015 to 2017 and had guttae and oedema confined to the central cornea, whilst the periphery was relatively unaffected [25]. All were combined with cataract procedures, where descemetorhexis of the central 4 mm of diseased DM was completed after phacoemulsification [25]. The average post-operative acuity via pinhole was logMAR 0.16 ± 0.09 following DMEK, and 0.13 ± 0.10 following DWEK (P = 0.44); however, the average time to reach 20/40 vision was 2.2 ± 2.8 weeks and 7.1 ± 2.7 weeks, respectively (P < 0.01) [25]. This suggests DWEK has equivalent visual outcomes to DMEK (the current standard of care), despite DWEK yielding a longer recovery time [25]. Of great importance is the fact that 53% of the DMEK patients had adverse events (e.g., increased intraocular pressure, inflammation of the anterior chamber, non-adherence of the graft, the need for anterior chamber paracentesis, or the need for a "rebubbling" procedure) [25]. In contrast, the DWEK cohort had no adverse events (P < 0.01), as well as no required extra procedures, donor tissue, or long-term immunosuppression [25].

Therefore, this implies DWEK is an effective and arguably preferable procedure to DMEK, as it is less likely to result in complications and it removes the burden of obtaining corneal donors and hence needing immunosuppression (which in itself can have substantial side effects). However, one cannot consider the sequalae alone and must also recognise other complexities surrounding DWEK, such as meticulous patient selection. Some of the mentioned advantages and disadvantages of DSAEK, DMEK, and DWEK (DSO) are summarised in Table 1 [8]. Encouragingly, ROCK inhibitors have already demonstrated the promotion of corneal endothelium wound healing and regeneration in animal models [8,13,17,26]. Some researchers have supplemented this with data from human subjects; for example, in 2019, a prospective study of 18 patients who underwent DWEK found that those assigned to post-operative ripasudil recovered vision more quickly and had a statistically significant greater average endothelial cell density at three, six, and 12 months [26]. This showed that ripasudil could increase cell density and expedite corneal clearance when contrasted with DSO without a ROCK inhibitor [8,26].

**Table 1:** Surgical techniques for the management of Fuchs endothelial corneal dystrophy. Reformatted after being sourced by open access permissions from Blitzer et al. [8]. The table describes the advantages and disadvantages of surgical techniques for the management of FECD. PK, DSAEK, DMEK, and DSO have already been described. FECD: Fuchs’ endothelial corneal dystrophy, PK: penetrating keratoplasty, DSAEK: Descemet’s stripping automated endothelial keratoplasty, DMEK: Descemet's membrane endothelial keratoplasty, DSO: Descemet's stripping only.

Technique	Advantages	Disadvantages
DSAEK	Eliminates "open sky” risk compared to PK	Requires indefinite immunosuppression
	Less post-operative astigmatism than PK	Acuity may be limited by host-donor stroma interface and higher-order aberrations
DMEK	Improved visual outcomes	Technically difficult to perform
	Fastest corneal clearance	Graft rejection remains a risk
DSO	No introduction of donor tissue	Requires careful patient selection
	Technically simple to perform	Long-term viability studies are ongoing

Once the general consensus that ROCK inhibitors can be effective is reached, the next stage is to investigate the best timing of administration, e.g., immediate application versus delayed. A pilot study as recent as 2021 further scrutinised the corneal clearance time following topical netarsudil (another ROCK inhibitor) after DWEK with cataract surgery for 10 FECD patients [27]. The trial involved all 20 eyes, where each patient’s first eye had immediate netarsudil post-operatively until corneal clearance, whereas the second eye had netarsudil withheld for two weeks after the corneal clearance time of this first eye (and only given if corneal oedema still remained) [27]. The average time taken to achieve corneal clearance was 4.6 ± 1.7 weeks in the first eye, a significantly shorter time than the 8 ± 1.9 weeks (P < 0.01) in the second eye not given immediate netarsudil [27]. The authors noted that corneal clearance developed between one and two weeks following netarsudil “rescue” therapy, concluding that these drops significantly reduced the time to corneal clearance after DWEK [27]. Moreover, the significantly greater endothelial cell count in eyes treated immediately using netarsudil in contrast to the eyes given delayed treatment insinuated that the prompt peri-operative stage is paramount in endothelial cell regeneration and movement [27]. 

Albeit being low-grade evidence from 2013, one of the first cases of late-onset FECD, initially referred for keratoplasty, is supportive of the above [28]. Instead of keratoplasty, a 52-year-old Japanese man with guttae and severe central corneal oedema in the left eye was treated with a topical ROCK inhibitor after trans-corneal freezing of the damaged endothelial cells [28]. Treatment with endothelial denudation and one week of the selective ROCK inhibitor Y-27632 enabled BCVA in the left eye to improve from 20/63 to 20/20 two weeks following treatment and then to 20/16 at six months [28]. This vision and good endothelial function continued 24 months after treatment [28]. The central corneal thickness significantly decreased below its pre-therapy level, and the patient had a complete recovery of his corneal clarity [28]. Such reported cases are extremely encouraging indicators that FECD treatment may progress into non-surgical approaches in the years to come.

Future considerations

Evidently, topical ROCK inhibitors have shown promising preliminary results in recent years as an adjunct to DWEK; and randomised placebo-controlled trials are currently in progress to investigate this further [8,11]. It is believed that the positive effects of ROCK inhibitors on central oedema in FECD will considerably contribute to innovative methods of treating corneal endothelial dysfunction in the future [17]. Whilst continued research into the role of ROCK inhibitors is indicated, DWEK itself requires more evaluation as a relatively novel technique, inclusive of ascertaining the optimal patient characteristics for a successful procedure [8]. ROCK inhibitors are also being explored as medical therapy in isolation to explore their potential in corneal clearance in the absence of DWEK surgery, yet our present knowledge of FECD pathogenesis has suggested this is possibly limited [8]. 

Another recent development has been the combination of ROCK inhibitors with cultured endothelial cells (CECs). These CECs can either be transplanted as a sheet or instilled as a solution into the anterior chamber [14,29]. A 2018 review described that a sizable hindrance to injected CECs was achieving proper adherence of these cells to the posterior cornea and preventing their transformation into a fibroblastic phenotype (which has been achieved in rabbit and monkey models by adding ROCK inhibitors, hypothesised to manipulate cell properties) [13,30]. In animal models, ROCK inhibitors enhanced cell adhesion and proliferation, promoted the expression of sodium-potassium ATPase and "ZO-1" proteins involved in endothelial function (so cells could self-organise and function appropriately), and prevented the apoptosis of the primate CECs [8,13,17,29,30]. Consequently, the use of ROCK inhibitors with CECs has provided a platform for the regenerative medical treatment of endothelial dysfunction. Additional ethically appropriate trials are needed to adequately determine the response in humans. Kinoshita et al. illustrate the preparation process of human CECs [31].

A first-in-human clinical trial reported in the New England Journal of Medicine (NEJM) in 2018 reported successful management of pseudophakic bullous keratopathy via intracameral injection of both CECs and ROCK inhibitors [31,32]. In the majority of the 11 participants, this therapy resulted in excellent visual acuity, increased endothelial cell density, reasonable reduction in central corneal thickness, resolution of corneal oedema, and maintained corneal clarity two years later, although they concluded more research is needed to investigate the efficacy of combining CECs with ROCK inhibitors for FECD [8,13,31,32]. Only one patient had a raised intraocular pressure of 27 mmHg eight months post-injection, which was diagnosed as glucocorticoid-induced glaucoma and resolved after trabeculotomy alone [31]. Due to this finding, if future trials were to be performed, the authors reported they would reassess the number and density of the cells injected [31]. Importantly, they recognised that the study design limited the ability to discern the extent that each factor contributed to the final clinical outcomes, identifying a critical area for upcoming studies to address [31]. The safety and efficacy of this new cell injection therapy were confirmed up to five years later [33]. A momentous advantage of CECs is that one corneal donor can potentially provide treatment for numerous patients, as seen in the NEJM trial, where cells obtained from seven independent young deceased donors were cultured and each of the 11 patients received an injection of cells from only one donor [31]. This could reduce the burden and potential costs of obtaining sufficient corneal donors, thus improving resource efficiency and long-term sustainability. It is yet to be determined exactly how many patients can benefit from a single corneal donor using this method.

Whilst these purely pharmacological therapeutic aspirations are extremely admirable, one should consider them alongside the substantial surgical advancements that are currently being explored, which could also increase the bioavailability of donor cornea. For instance, the first case series of quarter-DMEK has recently been published and is described as a potential hybrid procedure combining traditional circular DMEK with DWEK (DSO) [34]. In this prospective interventional series, quarter-DMEK was performed in 12 eyes (from 12 patients with FECD), where one quadrant of a full-diameter DMEK graft was transplanted [34]. They observed that BCVA at six months was similar to that expected of traditional DMEK but noted quite a large decline in endothelial cell density in the first month (possibly due to more extensive endothelial cell movement and measurement errors at the graft periphery), and that one-third of cases required "rebubbling" procedures by two months [34]. They advised that if the effects in the long run were found to coincide with conventional DMEK, then the quarter-DMEK approach has the potential to increase the grafts available four-fold [34]. Similar techniques are also being investigated, such as hemi-DMEK (semi-circular DMEK). Soh et al.’s flowchart summarise the current and experimental therapeutic options for FECD management [10]. The discovery of genotypes implicated in FECD has led to the quest to create gene editing strategies, which may be able to prevent the progression of the FECD phenotype but are unlikely to successfully remove guttae once they have formed in more advanced diseases [10].

## Conclusions

FECD constitutes progressive corneal endothelial dysfunction, where the endothelium loses its ability to maintain adequate deturgescence, resulting in corneal oedema, accumulation of guttae deposits in the DM, blurred vision, and eventually blindness if untreated. Keratoplasty is the only current definitive treatment. Through this review, we have discussed different treatment options for FECD over time, and have explored the advantages and constraints of these. The initial full-thickness corneal transplant (PK), although well-established and more reliable, has been superseded by EK (which has become the gold standard). PK has still proved necessary, since it is preferable for some patient characteristics, and may be required in severe FECD or as a secondary operation when other surgeries have been ineffective. Despite the lack of definitive data on which procedure is most superior, limited evidence has shown better BCVA following DMEK rather than DSAEK or PK. Generally, DSAEK has significantly less post-operative astigmatism than PK, as well as greater wound stability, fewer suture-related complications, fewer HOAs, better contrast sensitivity, faster visual rehabilitation, and a greater vision-related quality of life. DMEK has recorded higher patient satisfaction than DSAEK and has a lower risk of transplant rejection due to no additional foreign stroma, but DMEK has resulted in more graft dislocations requiring "rebubbling" than DSAEK. Furthermore, DMEK is technically more difficult to perform than DSAEK, since the absence of the adherent stroma poses challenges in preparing the donor tissue, as well as during insertion and surgical manipulation of this delicate donor endothelium. This has hindered the adoption of DMEK in the past compared to DSEK. However, the additional stromal tissue in DSAEK leads to more backscattering and more HOAs than DMEK, and so current perceptions are that the thinner graft in DMEK results in better visual outcomes. Although DMEK is regarded as the current standard of care, some studies have claimed equivalent visual outcomes to DSO or DWEK (though the latter yields a longer recovery time, and is only advisable for mild to moderate central FECD). Whilst DMEK can lead to adverse events including graft non-adherence, inflammation, and increased intraocular pressure, DWEK boasts minimal graft concerns due to no donor tissue being transplanted, and hence no long-term immunosuppression is needed either.

DWEK relies on healthy peripheral endothelial cells migrating centrally, sufficiently repopulating the endothelium, and thereby restoring the pump function, corneal deturgescence, and therefore vision. Topical ROCK inhibitors after DWEK can increase endothelial cell density and expedite corneal clearance compared to DWEK alone. This is because ROCK inhibitors allow the regulation of cell migration, proliferation, and apoptosis. Although lower-quality general consensus data exists, there is a considerable insufficiency of evidence-based RCTs for FECD management, possibly impeded by ethical constraints and the need for long-term follow-up of clinical outcomes. Nevertheless, the future appears very bright for FECD management. Experimental methods have already demonstrated efficacy, such as pharmacological cell-based regeneration therapy via ROCK inhibition, with or without intracameral CEC injections. These minimally-invasive techniques pledge a plethora of benefits, including decreased graft rejection than with keratoplasty, excellent visual acuity, resolution of corneal oedema, reduced burden and costs of obtaining sufficient corneal donors, and greater efficiency in that CECs from only one donor can treat multiple patients. It remains unclear exactly how many patients can be treated by a single corneal donor via this method. By the same token, quarter-DMEK surgery can quadruple the bioavailability of donor cornea, whilst heralding similar visual outcomes to conventional DMEK; however, more large-scale trials are needed. Since FECD has been associated with oxidative stress, especially in the mitochondria, this has suggested another potential treatment target. Gene editing strategies are also being investigated for early diseases. Nonetheless, we do not feel these new options will render keratoplasty completely redundant, especially in severe FECD, but believe they may be an adjunct or perhaps even a contemporary gold standard. Overall, as necessity is the mother of invention, we expect scientific progress will continue to adapt to our current knowledge of FECD, and we project that more innovative methods to treat FECD will be created in the future that are more sustainable, cost-effective, and generate superior clinical outcomes.
